# Epicardial Termination of Left Atrial Appendage Atrial Tachycardia

**DOI:** 10.19102/icrm.2021.121004

**Published:** 2021-10-15

**Authors:** Marco Polselli, Filippo M. Cauti, Pietro Rossi, Riccardo Maddalena, Stefano Bianchi

**Affiliations:** ^1^Arrhythmology and Electrophysiology Unit, Ospedale San Giovanni Calibita, Fatebenefratelli Hospital, Isola Tiberina, Rome, Italy

**Keywords:** Case report, epicardial ablation, left atrial appendage tachycardia

## Abstract

This case report describes a third successful attempt to ablate a focal atrial tachycardia originating from the left atrial appendage in a highly symptomatic 49-year-old woman using a combined endocardial–epicardial approach, which could be taken into consideration as a safe and effective alternative method for treating similar arrhythmias originating from complex sites.

## Case presentation

A 49-year-old woman with a history of incessant atrial tachycardia (AT) was referred to our arrhythmia unit. She was diagnosed with focal AT at the age of 17 years and treated with different classes of antiarrhythmic drugs, without a significant burden reduction. She was found to be intolerant to flecainide because of vision disturbances. Therefore, 160 mg/day of sotalol was prescribed, which she continued to take despite experiencing hypotension and asthenia as side effects.

After multiple electrical cardioversions, she was referred for a first ablation attempt at another center. The activation map revealed a left atrial appendage (LAA) focal AT, with a cycle length (CL) of 485 ms (124 bpm). Radiofrequency (RF) ablation was performed from the endocardium, near the entrance of the LAA, without leading to AT termination. She was thus rescheduled for cryoablation in another center, in order to avoid LAA perforation. A second attempt was made with the use of a cardiac cryoablation catheter (Freezor; Medtronic, Minneapolis, MN, USA), which led only to a slowing of the arrhythmia, without termination. The patient was then referred to our department for re-evaluation.

**Figure 1:**
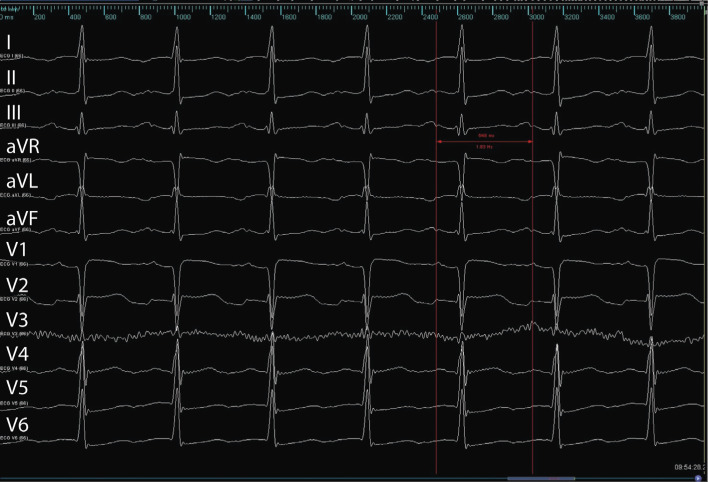
Baseline ECG showing the clinical atrial tachycardia, with a CL of 548 ms (109 bpm). Negative P-waves in D1 and positive P-waves in DII, DIII, and precordial leads.

The admission electrocardiogram (ECG) showed an incessant AT with a CL of 548 ms (109 bpm), with negative P-waves in D1 and positive P-waves in DII, DIII, and precordial leads **(Figure 1)**. Cardiac magnetic resonance imaging was performed to study the shape of the LAA and its anatomical details, which demonstrated a “chicken wing” morphology. A new ablation procedure was scheduled with a combined endocardial–epicardial approach.

**Figure 2:**
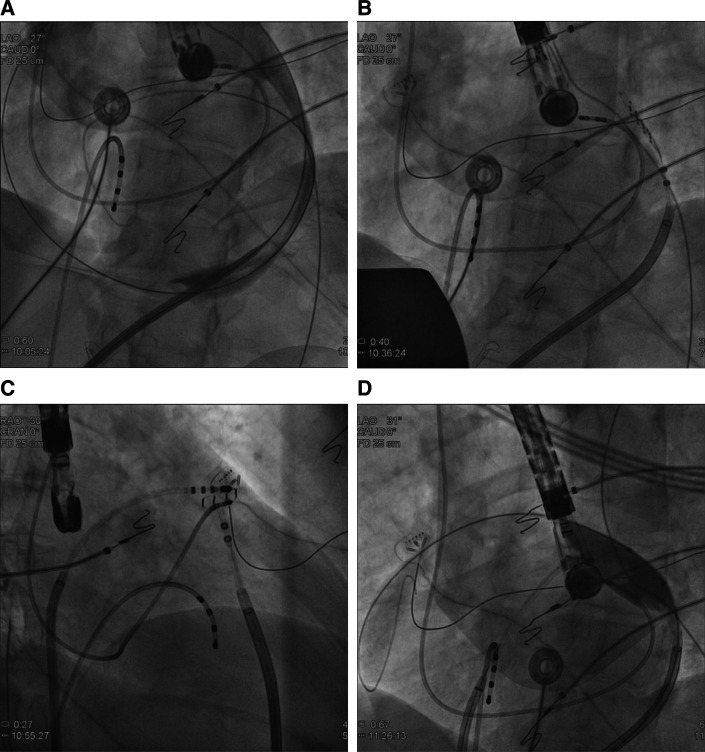
**A:** Epicardial access confirmed via dye injection. **B and C:** Left and right anterior oblique fluoroscopic views of the LAA and catheter positioning. **D**: Epicardial balloon inflated to displace the phrenic nerve during ablation.

Under general anesthesia, a transesophageal echocardiography probe was positioned to rule out LAA thrombi, monitor catheter positions, and control RF delivery. Before systemic anticoagulation, inferior epicardial access was obtained to target epicardially the region of interest via a steerable sheet (Agilis EPI; Abbott) **(Figure 2A)**. The activated clotting time was maintained between 300 and 350 seconds. A high-density electroanatomic map (EAM) of the epicardial layer above the LAA was obtained via a multipolar high-density catheter (HD Grid; Abbott, Chicago, IL, USA) **(Figures 2B and 2C)**. High output pacing and tag positioning on the EAM outlined the phrenic nerve course.

A left atrial endocardial EAM was obtained using the HD Grid, via a patent foramen ovale, with the catheter’s soft, closed shape permitting gentle reconstruction of the LAA endocardial anatomy. The LAA EAM was then refined via an optical fiber, contact-sensing, open-irrigated ablation catheter (TactiCath SE, Sensor Enabled; Abbott), which was constantly monitored via transesophageal echocardiography visualization.

**Figure 3:**
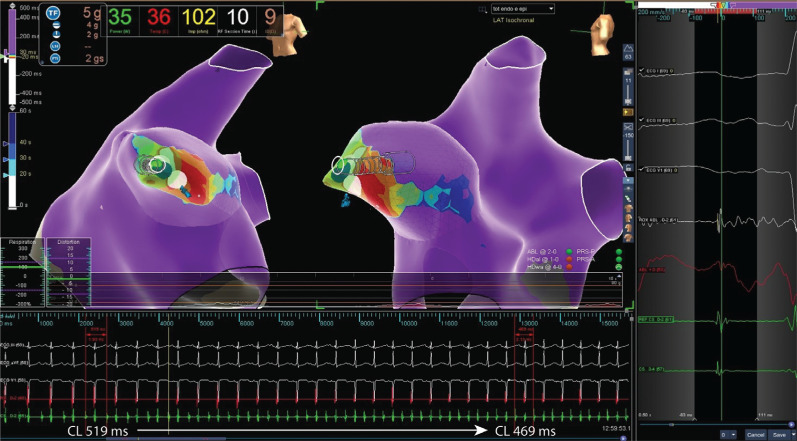
An endocardial attempt with arrhythmia warm-up.

**Figure 4:**
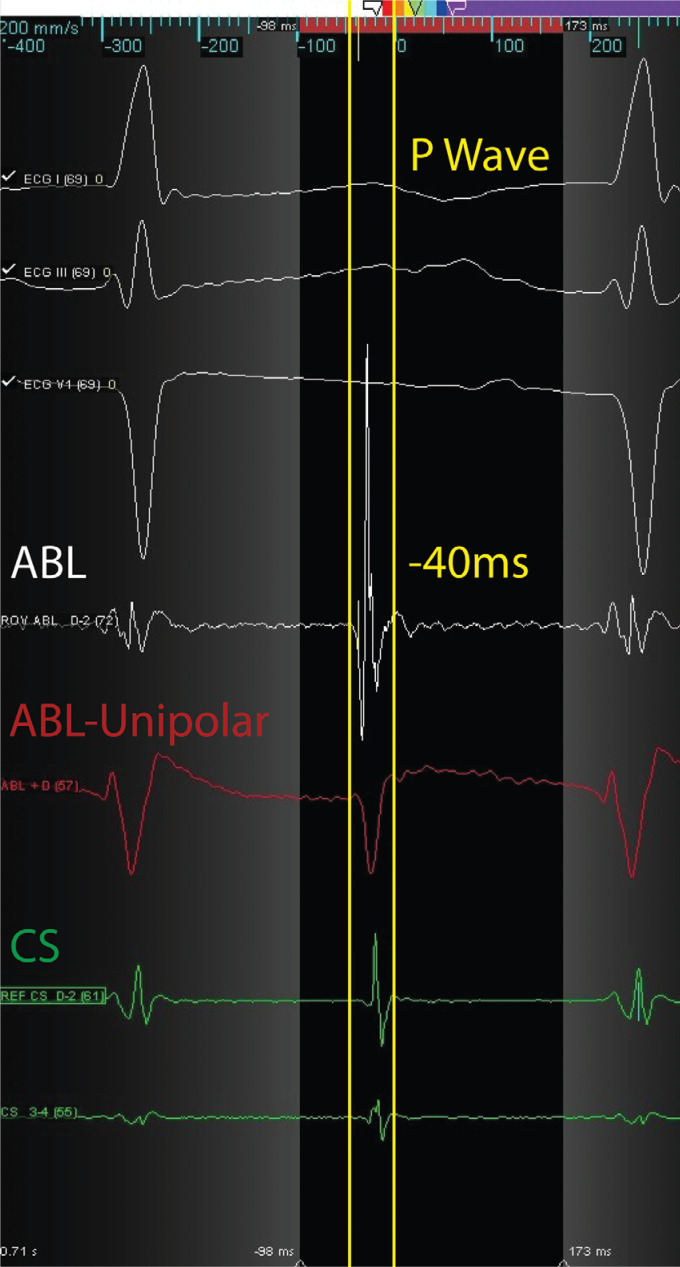
Epicardial EGM recorded by the ablation catheter. Unipolar EGM with QS activation. ABL: ablation; CS: coronary sinus; ECG: electrocardiogram.

**Figure 5:**
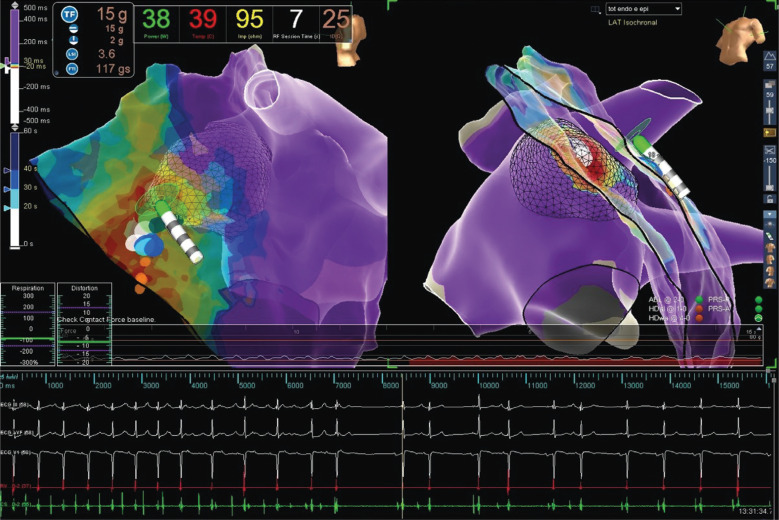
Epicardial interruption during RF of the clinical tachycardia. Ablation parameters: power of 38 W, temperature of 39°C.

Two endocardial RF applications were performed with 35 W at 43°C at the earliest electrogram (EGM) after phrenic nerve displacement via an epicardial balloon **(Figure 2D)**. The RF application influenced the arrhythmia cycle by warming it up (CL shortened), without interrupting it **(Figure 3)**. A second attempt was then made epicardially, during phrenic nerve displacement via a second epicardial access, at the earliest EGM (−40 ms), slightly lower and more anterior in respect to the endocardial ablation site **(Figure 4)**. A single RF pulse at 40 W and 43°C (15 g of tangential force) terminated the arrhythmia after seven seconds **(Figure 5)**.

An intrapericardial corticosteroid was injected as per the center’s protocol at the end of the procedure and a pigtail catheter was left in the left lateral epicardial wall. No complication occurred. Epicardial drainage was removed three hours after the procedure. No more arrhythmias occurred during the hospital stay. The patient was discharged the day after the procedure without antiarrhythmic drugs. Considering the pinpoint endocardial application and the LAA filling/voiding speeds, anticoagulation therapy was maintained for one month after the ablation procedure.

At three months of follow-up, a 24-hour Holter ECG was performed, which showed the absence of arrhythmias, excluding a small number of isolated premature atrial complexes. The patient was completely asymptomatic and reported a drastic improvement in her quality of life.

## Discussion

The LAA is among the specific anatomic structures that can be responsible for AT originating from the left atrium. This structure originates from the embryonic left atrium^1^ and usually has an elongated, finger-like structure with different shapes forming its body (“chicken wing,” “cactus-like,” “cauliflower,” and “windsock”)^2^ differently from the right atrial appendage, which has a more constant, triangular shape. The LAA is positioned between the left superior pulmonary vein, the left circumflex artery, and the great cardiac vein. The LAA anatomy, with different shapes; pectinate muscles; and the fragile, thin myocardium being among its characteristics, poses some serious challenges to the electrophysiologists approaching this structure, making it difficult to easily reach the distal part of the appendage with catheters or making it risky to ablate with sufficient energy inside this structure.

ECG patterns have been developed^3–6^ to identify the specific site of origin of a focal tachycardia, but the targeting capability of a modern high-definition mapping system overcomes most of the uncertainties about the exact electroanatomic source of the tachycardia, guiding the operator to the precise site of origin to target for ablation.

Our case report underlines the relevance of these little “gaps” in the precise localization of the ectopy inside particular and anatomically complex structures, where ablation could prove to be too dangerous and, in some cases, harmful due to the low thickness of the myocardium. In such cases, where ectopy arises from the LAA, an epicardial approach could be the only way to reach and effectively ablate the source of the arrhythmia, when different ablation strategies fail (endocardial RF and endocardial cryo in our case), as reported in a few previous experiences.^7,8^ Moreover, for this same reason, an epicardial approach could prove counterintuitively safer than an endocardial approach, limiting the power and application time to those strictly necessary to ablate the ectopic focus, thus preserving the integrity of the surrounding myocardium and structures.

In our case, we were able to map and then displace the phrenic nerve with a balloon inflated in the pericardium, avoiding contact between the ablating catheter and the nerve, thereby eliminating the chance of palsy.

Different approaches for LAA tachycardia ablation have been proposed, such as cryoballoon LAA isolation,^9^ LAA resection with thoracoscopic or video-assisted thoracoscopic surgery,^10,11^ or device-based approaches (eg, Atriclip; Atricure, Mason, OH, USA^12^ or Lariat; SentreHEART, Redwood City, CA, USA^13^), all with relevant potential risks (eg, staple line failure).

An endocardial–epicardial approach could also help in reducing the hospital stay and the associated costs.

In terms of safety, a confined, short RF application should not reduce LAA systolic function or total blood flow, as previously demonstrated.^5,14^

## Conclusions

Epicardial ablation is an effective and safe approach to ablate focal tachycardias originating from the LAA, particularly from the distal part of it, which can be difficult to reach with an endocardial catheter, given the complex anatomy^1^ of the structure and the frailty of the myocardium at this level. This minimally invasive approach is probably safer than others, like appendage resection (risk of suture failing; furthermore, the LAA is not a useless structure), whole isolation of LAA with devices (presence of a foreign body), or cryoablation^15^ (possibility of clot formation and LAA contractility reduction), and could help to avoid ineffective and eventually harmful multiple endocardial attempts.^16,17^
